# Successful combination of Rituximab and plasma exchange in the treatment of cryoglobulinemic vasculitis with skin ulcers: a case report

**DOI:** 10.4076/1757-1626-2-7859

**Published:** 2009-07-06

**Authors:** Tiziano Tallarita, Massimiliano Gagliano, Daniela Corona, Giuseppe Giuffrida, Alessia Giaquinta, Domenico Zerbo, Massimiliano Sorbello, Pierfrancesco Veroux, Massimiliano Veroux

**Affiliations:** 1 Department of Surgery, Transplantation and Advanced Technologies; Vascular Surgery and Organ Transplant Unit, University Hospital of CataniaVia Santa Sofia, 86 95123 CataniaItaly; 2 Department of Surgery, Transplantation and Advanced Technologies; Anaesthesia and Intensive Care Unit, University Hospital of CataniaVia Santa Sofia, 86 95123 CataniaItaly

## Abstract

**Introduction:**

Type II mixed cryoglobulin syndrome is a systematic vasculitis mainly linked to immune complex deposition in several organs and to hepatitis C virus infection. Therapeutic strategies can target either the viral trigger hepatitis C virus if present, or pathogenic events downstream the triggering infection, e.g, the proliferation B-cells directly. Antiviral therapy should be considered as a first-line treatment in many HCV-positive patients. However, it may prove ineffective, contraindicated, or poorly tolerated. The other available treatment [such as cytotoxic agents, steroids] may lead to life-threatening complications and may be difficult to manage in the long term.

**Case presentation:**

We report on a 75-year-old patient with long-lasting hepatitis C virus infection [18 years], a long-lasting cryoglobulinemia [7 years] resistant to common antiviral therapy, diabetes mellitus and deep skin ulcers, successfully treated with the combination therapy of Rituximab and plasma exchange.

**Conclusion:**

Plasma exchange in combination with Rituximab may be useful to heal skin in those patients who are non responsive to Rituximab alone, by avoiding a leg amputation.

## Introduction

Type II mixed cryoglobulinemia (tIIMC ) is a systemic vasculitis caused by immune complexes formed by monoclonal IgM rheumatoid factor and polyclonal IgG [1]. In the large majority of cases, this disorder is caused by chronic hepatitis C virus infection [2]. The immunological mechanism underlying tIIMC consists on the HCV-driven monoclonal expansion of a subset of CD20+, CD27+ memory B-cells expressing a characteristic cross-idiotype [3]. Therefore, the therapy of tIIMC can be focused on inhibition of viral replication or on inhibition of B-cell proliferation. Eradication of HCV infection by antiviral treatment usually leads to the disappearance of cryoglobulins and the regression of vasculitis [4,5], but several patients are resistant to antiviral therapy or cannot be treated because of side effects or contraindications. Recently, anti B cell therapy with rituximab has been proposed as salvage treatment for patients failing to respond to antiviral therapy [6-11]. Rituximab (RTX), a mouse/human chimeric monoclonal anti-CD20 antibody, selectively targets the B-cell compartment from B-cell step to plasma cells, and results in prolonged depletion of normal B-cells from peripheral blood [12]. RTX is approved for the treatment of non-aggressive non-Hodgkin lymphoma [13]. Given the good safety profile and the role of B-cells as antigen-presenting cells or pathogenic antibody producers in many autoimmune disorders, RTX has been used off-label for numerous patients suffering from a great variety of autoimmune diseases [14,15].Therefore RTX has been successfully employed in tIIMC patients, including HCV-negative and alpha-interferon resistant patients. We report a successful treatment of a patient with cryoglobulinemic vasculitis presenting with skin ulcers, with a combination of rituximab and plasma exchange.

## Case presentation

**Figure 1.**
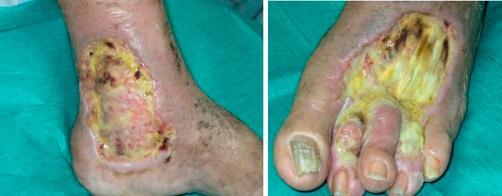
Ulcer to the leg extending to the medial **(A)** and the anterior **(B)** part of the foot.

**Figure 2.**
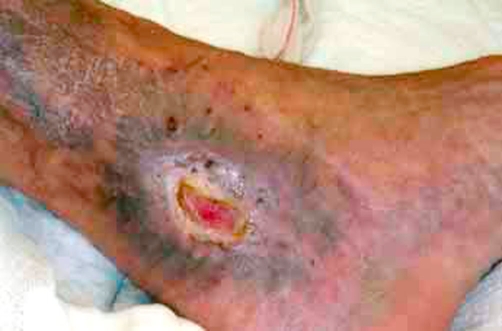
Near-complete resolution of the ulcer of the medial part of the foot 4 months after treatment.

A 75-year-old Caucasian Italian man was admitted because of ulcers on left leg. His past medical history was relevant for a long-lasting (18 years) HCV infection and a long-lasting (7 years) tIIMC resistant to antiviral therapy. He had been undergone an antiviral therapy with a combination of alpha interferon, Ribavirin and prednisolone, with a significant reduction of HCV activity, as demonstrated by a reduction of HCV-RNA levels (800000 UI/L to 13000 UI/L), but without reduction of cryocrit and B-cells (cryocrit 93/μL and B-cells 286/μL). After two years of therapy discontinuation, the patient was admitted at our unit. The HCV activity, cryocrit and B-cell levels were very high (800000 UI/L, 132/μL, 412 B-cells/μl respectively). The leg ulcers measured 8 × 6 cm and 10 × 5 cm and were very aching with signs of infection (Figure 1). We started an antiviral therapy with prednisolone, Interferon-α and Ribavirin which brought to a important reduction of HCV activity (from 800000 UI/L to 54000 UI/L) with slight reduction of cryocrit (132/μL to 112/μL) and B-cells (412/μL to 338/μL). After two weeks of treatment there was no significant improvement in ulcer’s healing so we started a therapy with Rituximab. We administered a single course of RTX at a dose of 375 mg/m^2^ weekly for 4 weeks, and one course of 375 mg/m^2^ every two months as maintenance regimen. Our patient showed a mild reaction during the first infusion of RTX (mild hypotension and tremor) easily controlled with steroids administration. Treatment with RTX resulted in a slight reduction of cryocrit and B-cells, without significant improvement on ulcer’s healing. Finally, the patient underwent five sessions of plasma exchange to reduce cryocrit and a dose of RTX of 375 mg/m^2^ was administered. This treatment resulted in reduction of cryocrit (2 /μL) and B-cells (205 /μL) and the skin ulcers improved with important reduction of pain. The skin ulcers completely recovered in 6 months (Figure 2).

## Discussion

Type II mixed cryoglobulinemia is the result of monoclonal IgM paraprotein with rheumatoid activity binding to polyclonal IgG in the circulation, causing immune complexes, complement activation, vasculitis, and gromerulonephritis. tIIMC often is associated with chronic HCV infection.

Patients with a long -lasting tIIMC often are non responders to antiviral therapies. Our patient was unsuccessfully treated with antiviral therapy and Rituximab, and only a combination with RTX and plasma exchange resulted in a clinical improvement. We may speculate that plasma exchange therapy may have removed some immune complexes that can bind part of RTX, and significantly reduced cryocrit and rheumatoid factor, by finally improving the efficacy of Rituximab.

## Conclusions

After the failure of RTX the only possibility to kill pain in a patient with skin ulcers is amputation. Plasma exchange in combination with RTX can be useful to heal skin in those patients who are non responsive to RTX alone, by avoiding a leg amputation. The mechanism remains unknown. Further studies will be useful to define if the combination of plasma exchange with RTX can be used in all patients unresponsive to RTX or antiviral therapies.

## Abbreviations

HCV: Hepatitis C Virus
IgG: Immunoglobulin G
IgM: Immunoglobulin M
RTX: Rituximab
tIIMC: Type II mixed cryoglobulinemia
